# The *In vitro* Effect of Nanoliposomal Amphotericin B Against *Rhizopus arrhizus* Isolated From COVID-19-Associated Mucormycosis Patients

**DOI:** 10.30699/ijp.2024.2033626.3320

**Published:** 2025-01-10

**Authors:** Ali Ahmadi, Sayed Jamal Hashemi, Seyed Mahdi Rezayat, Roshanak Daie-Ghazvini, Mahmoud Reza Jaafari, Jamileh Esmaeili, Fatemeh Saiedmohammadi, Farzaneh Afshari, Laura Alcazar-Fuoli, Alireza Abdollahi, Sadegh Khodavaisy

**Affiliations:** 1 *Department of Medical Parasitology and Mycology, School of Public Health, Tehran University of Medical Sciences, Tehran, Iran*; 2 *Students' Scientific Research Center, Tehran University of Medical Sciences, Tehran, Iran*; 3 *Food Microbiology Research Center, Tehran University of Medical Sciences, Tehran, Iran*; 4 *Department of Pharmacology, School of Medicine, Tehran University of Medical Sciences, Tehran, Iran*; 5 *Department of Pharmaceutical Nanotechnology, School of Pharmacy, Mashhad University of Medical Sciences, Mashhad, Iran.*; 6 *Nanotechnology Research Center, Pharmaceutical Technology Institute, Mashhad University of Medical Sciences, Mashhad, Iran*; 7 *Department of Pharmacology and Toxicology, Faculty of Pharmacy, Tehran University of Medical Sciences, Tehran, Iran*; 8 *Cancer Immunology Project (CIP), Universal Scientific Education & Research Network (USERN), Tehran, Iran*; 9 *Mycology Reference Laboratory, National Centre for Microbiology, Instituto de Salud Carlos III, Madrid, Spain*; 10 *Department of Pathology, Imam Khomeini Hospital Complex, Tehran University of Medical Sciences, Tehran, Iran*

**Keywords:** Nanoparticle Drug Delivery System, Liposome, Amphotericin B, Rhizopus oryzae, COVID-19, Mucormycosis

## Abstract

**Background & Objective:**

*Rhizopus arrhizus*, a major contributor to COVID-19-associated mucormycosis (CAM) globally. Nanoliposomal amphotericin B (NLAmB) presents a promising approach due to its enhanced drug delivery and reduced side effects. This study aimed to assess the *in vitro* antifungal susceptibility of NLAmB against *R. arrhizus* isolated from CAM patients.

**Methods:**

Thirty-nine *R. arrhizus* isolated from CAM patients were identified through phenotypic characterization, MALDI-TOF, and the internal transcribed spacer rDNA region (ITS) sequencing approaches. Antifungal susceptibility testing (AFST) for NLAmB, amphotericin B (AmB), posaconazole (PSC), and isavuconazole (ISC) was conducted through broth microdilution methods according to the European Committee on Antimicrobial Susceptibility Testing (EUCAST) standard E.DEF 9.4. Results were analyzed for MIC ranges, MIC50, MIC90, and distributions.

**Results:**

NLAmB demonstrated superior *in vitro* efficacy against *R. arrhizus* (MIC50/90, 0.063/0.25 μg/ml) compared to AmB, PSC, and ISC. PSC exhibited notable activity (MIC range: ≤0.031 - ≥16 μg/ml).

**Conclusion:**

The study emphasized NLAmB's sustained activity, making it a potential alternative to LAmB. Further exploration and clinical correlation are warranted to validate NLAmB in CAM treatment.

## Introduction

Mucormycosis is a serious invasive fungal infection (IFI) with a high mortality rate that usually affects immunocompromised individuals, which has become increasingly frequent in patients infected with severe acute respiratory syndrome coronavirus 2 (SARS-CoV-2) (1). COVID-19-associated mucormycosis (CAM) is also increasingly reported in India, Iran, and some other countries (2). *Rhizopus arrhizus* is the most frequent cause of mucormycosis globally (3). These infections are typically challenging to treat for a variety of reasons, including acute disease progression, angioinvasion, tissue necrosis, and delayed diagnosis, as well as primary resistance to many antifungal drugs currently available (4). The key to effective management and treatment strategies including addressing the underlying predisposing condition, surgical debridement, and prompt antifungal therapy, is early diagnosis (5). The first line of antifungal therapy is the intravenous liposomal form amphotericin B (LAmB), which is less nephrotoxic than conventional forms of amphotericin B (AmB) and can therefore be administered for longer periods. (6). The use of drug-delivery nanocarriers is another method for reducing the side effects of these drugs. Designing a system with adequate drug loading, ideal release qualities, a short half-life, and minimal toxicity is the aim of researchers in the synthesis and optimization of drug delivery (7). A nanocarrier carrying AmB attaches to the fungal cell and allows drug molecules to be released slowly into the fungus cell membrane (8). Because of their great efficiency, biocompatibility, and biodegradability, nanoliposomes are a viable alternative for capturing hydrophobic drugs. They might be used as a tactic to lessen side effects and improve drug treatment. Additionally, they are quite stable, easy to make, and store (9). Posaconazole (PSC) and isavuconazole (ISC), two additional antifungal medications, are also employed as step-down, salvage, or primary therapies when LAmB cannot be administered (10). The antifungals AmB, PSC, and ISC are typically effective *in vitro* against members of the order Mucorales. However, clinical response rates are still below average, and some isolates may exhibit reduced susceptibility to these agents (10,11). *In vitro* susceptibility testing is critical for improving our epidemiological understanding of mucormycosis, forecasting susceptibility patterns, conducting outbreak investigations, and providing clinicians with helpful information about medication appropriateness (12). Since AmB has poor stability and low solubility in water, we hypothesized that if the drug was loaded into the hydrophobic part of nanoliposomes, it would be released slowly after contact with fungal cells and its stability and solubility would be increased. The objective of this study was to evaluate the antifungal susceptibility profiles of a large collection of *Rhizopus arrhizus* isolated from CAM patients against nanoliposomal amphotericin B (NLAmB) in comparison with routine antifungal drugs such as AmB, PSC, and ISC. 

## Material and Methods

### Fungal Identification

A total of 39 clinical *R. arrhizus* isolated from CAM patients were included. Isolates were identified by their macroscopic and microscopic characteristics, MALDI-TOF MS VITEK MS (bio Mérieux, Marcy-l'Etoile, France), and internal transcribed spacer rDNA region sequencing. For the MALDI-TOF method, the isolates were identified using the MBT (Maldi Biotyper) filamentous fungi library. The recognition score was between 1.74 and 2.06. Low-scoring isolates were identified using sequencing and matched to the species level. For sequencing, we used the Genomic Extraction Kit (GeneAll, Korea) to extract the genomic DNA from cultures grown on Potato dextrose agar (PDA; Becton Dickinson, Sparks, MD, USA), in accordance with the manufacturer's recommendations. The D1/D2 domains of the large subunit ribosomal DNA (rDNA) genes and the internal transcribed spacer (ITS) rDNA region were amplified and sequenced (13). The sequences were then used to perform BLASTn searches in GenBank (https://blast.ncbi.nlm.nih.gov). Identification was defined by > 99.5% sequence similarity, and at least 90% query coverage (98 to 100% identity, E-value = 0.0).

### Preparation and Characterization of NLAmB

NLAmB was developed at Mashhad University of Medical Sciences, Iran as previously reported (14). Briefly, phosphatidylcholine and cholesterol were used to prepare liposomes containing AmB (Synbiotics Limited, Vadodara, India) (15). Using dynamic light scattering (Malvern, Nano-ZS, UK), the particle diameter of each sample was determined in triplicate. The liposomes' Zeta potential was determined by electrophoretic light scattering by a Malvern Zetasizer Nano ZS. Following the proper dilution with MOPS buffer (10 mM, pH 7.4), the measurement was carried out at 25°C. At least three repetitions were made of each measurement. The HPLC (Knauer, Germany) method was used to determine the concentration of AmB in nano-liposomal formulations, as previously mentioned (16). Safety evaluation of NLAmB has been previously described (17).

### Susceptibility testing

AFST for AmB, PSC (each from Sigma-Aldrich, St. Louis, MO, USA), ISC (Astellas Pharmaceuticals, Northbrook, IL, USA), and NLAmB (Exir Nano Sina, Tehran, Iran) was performed by broth microdilution methods according to the European Committee on Antimicrobial Susceptibility Testing (EUCAST) standard E.DEF 9.4 (18). Briefly, each drug's stock solutions were prepared by dissolving in dimethyl sulfoxide (DMSO), and further dilutions were made in RPMI 1640 with L-glutamine and phenol red, without bicarbonate, buffered with 0.165 M morpholine propane sulfonic acid (MOPS), supplemented with 2% glucose, pH 7.0, and with the final concentrations for each antifungal ranging from 0.031 to 16 μg/mL. After 24 hours of incubation at 35°C, MICs were visually read at 100% inhibition of growth, and each day of testing contained *A. fumigatus *ATCC 2004305 and *A. flavus *ATCC 204304 as quality control isolates. Epidemiological cutoff values (ECVs) ≥97.5% for AmB 4 μg/mL and PSC 2 μg/mL have been reported for *R. arrhizus*, but none for other antifungals are currently available (19). ECVs were used to categorize isolates as either wild-type (WT) isolates with no acquired resistance to the treatment under consideration or non-wild-type (NWT) isolates with decreased sensitivity and putative resistance mechanisms.

## Results

### Characteristics of NLAmB

**Fig. 1:**
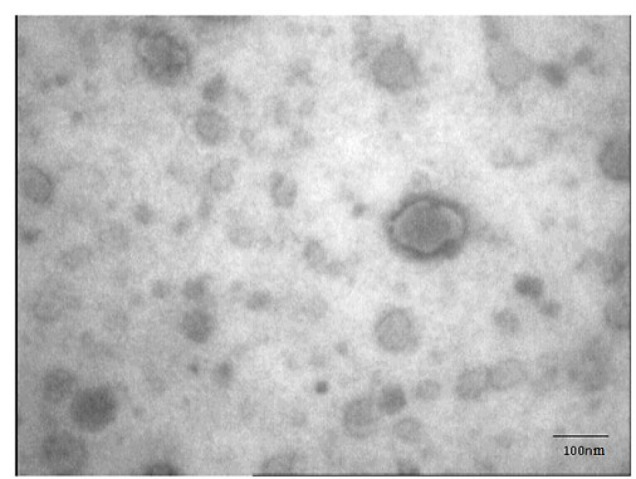
Transmission electron microscopy micrograph of NLAmB showing their round morphology

**Fig. 2:**
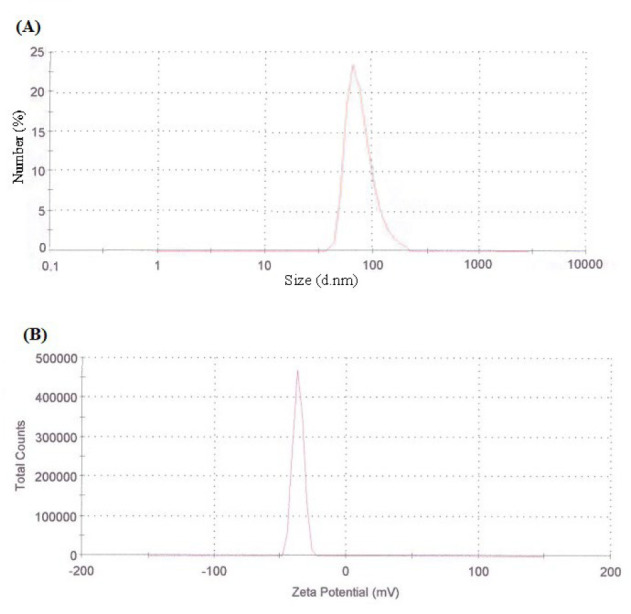
Characterization of NLAmB. (A) Particle size distribution and (B) zeta potential measurement using a Malvern Instruments Zetasizer Nano ZS. The measurement was performed in triplicate.

The shape and size of the developed NLAmB were analyzed by a transmission electron microscopy (TEM) and Zeta analyzer, respectively. The TEM micrograph study demonstrated that the NLAmB had a round morphology and an average particle diameter of approximately 100 nm. (Figure 1). The particle size and size distribution were evaluated by a Zetasizer and found to be 113.5±10.4 nm (Figure 2A). Additionally, the particle surface charge (Zeta potential) was −36.5±4.2 mV (Figure 2B). Control empty liposome containing DMSO with the same percent in NLAmB was inactive against *R. arrhizus.* The size and zeta potential of the Control empty liposome were determined to be 106.3±2.1 nm and −32.9±4.59 mV. 

### Antifungal Susceptibility Testing

The results of the *in vitro* AFST of 39 isolates of *R. arrhizus *are shown as MIC ranges, MIC50, MIC90, geometric mean (GM), and MIC distributions for NLAmB, AmB, PSC, and ISC in Table 1. Overall, NLAmB (MIC_50/90_, 0.063/0.25 μg/mL) was the most effective antifungal against *R. arrhizus* followed by AmB (MIC_50/90_, 0.5/4 μg/mL). 

Among the azoles, PSC (MIC range = ≤0.031 - ≥16 μg/mL) was the most active, while ISC (MIC range = 0.5 to ≥16 μg/mL) had the highest MICs. High MICs (≥4 μg/mL) were present in 13% (5/39), 41% (16/39) and 69% (27/39) of the isolates for AmB, PSC and ISC, respectively. While against all isolates, NLAmB demonstrated low MICs (≤1 μg/mL). According to the available ECV present in tests with *R. arrhizus*, we concluded in this study that all isolates were WT for NLAmB and 31 NWT isolates for AmB (n= 5) and for PSC (n = 26).

## Discussion

The clinical manifestations of COVID-19 are diverse, ranging from nonspecific or asymptomatic flu-like symptoms to pneumonia, sepsis, and life-threatening complications such as multiple organ failure and acute respiratory distress syndrome (ARDS). In addition, secondary fungal and bacterial infections have been identified as key risk factors for unfavorable COVID-19 outcomes (20). Recently, multiple case reports have documented mucormycosis in patients with SARS-CoV-2 infection (21). Reported mortality rates for CAM patients vary significantly in the literature, ranging from 14% to 87.5% (21,22), and many survivors experience vision loss due to rhinocerebral disease (22,23).

**Table 1:** In vitro activity of NLAMB and three comparators antifungal agents against 39 clinical *R. arrhizus* isolates.

Antifungal drugs			MIC Range				MIC _50_			MIC _90_			GM	
NLAmB			≤0.031 to 1				0.063			0.25			0.085	
AMB			0.25 to 8				0.5			4			0.808	
PSC			≤0.031 to ≥16				2			16			2.303	
ISC			0.5 to ≥16				4			16			4.145	
MICs (μg/ml)														
	**≤0.031**	**0.063**		**0.125**	**0.25**	**0.5**		**1**	**2**		**4**	**8**		**≥16**
NLAmB	11	**11**		5	9	2		1						
AMB					4	**19**		7	4		4	1		
PSC	1				1	4		7	**10**		7	1		8
ISC						2		4	6		**15**	2		10

Abbreviations: NLAMB, nanoliposomal amphotericin B; AMB, amphotericin B; PSC, posaconazole; ISC, isavuconazole. Numbers in bold are modal value.

Mucorales are inherently resistant to several antifungals, including VRC and the echinocandins, thus limiting treatment options (24). Higher doses of LAmB are frequently required, and PSC and ISC can be used as alternatives or for transitioning to oral therapy. Whenever feasible, antifungal therapy should be combined with surgical debridement and addressing any underlying immunological deficits or other risk factors (24). Using a nanocarrier improves drug stability and pharmacokinetics, reduces adverse effects, and enhances therapeutic benefits; nanocarriers are also relatively easy and cost-effective to prepare, store, and transport (25).

Among all antifungals tested in this study, NLAmB displayed the strongest and most sustained in vitro activity, followed by AmB and PSC. Previous studies have shown NLAmB’s antifungal efficacy against clinical strains of *Trichophyton interdigitale* and *Trichophyton rubrum* isolated from onychomycosis (26) and against vaginal candidiasis in animal models (27). However, its antifungal activity against invasive mold fungal pathogens has not yet been explored. Overall, except for ISC, the results of this investigation are consistent with previous findings for AmB and triazoles against Mucorales (28–32). The increased MIC of ISC observed in our study could be attributed to long-term antifungal prophylaxis in COVID-19 patients, aligning with studies of *R. arrhizus* isolated from COVID-19 patients (33,34).

Despite being one of the largest in vitro AFST studies of *R. arrhizus*, certain limitations should be noted. First, because clinical outcome data were unavailable, we could not correlate our in vitro susceptibility results with treatment failures or responses. Second, neither the Clinical and Laboratory Standards Institute (CLSI) nor EUCAST has set antifungal clinical breakpoints for any Mucorales. As such, it is unclear how the MIC data—particularly the higher values observed in this study—would translate clinically. While epidemiological cutoff values (ECVs) for AmB, PSC, and itraconazole (ITC) have been reported for certain Mucorales, including *R. arrhizus*, no ECVs currently exist for other antifungals (19). Lastly, these results reflect only the cultured *R. arrhizus* strains from CAM patients in Iran and may not fully represent the AFST status of other mucormycosis agents.

## Conclusion

Our findings showed higher MIC values than those reported elsewhere, suggesting COVID-19 may influence the AFST profiles of *R. arrhizus* isolates from CAM patients. When timely fungal identification is not possible, or in cases where microbiological failure may be contributing to poor clinical outcomes, AFST of Mucorales isolates could be warranted. This study highlights the sustained activity of NLAmB and suggests that, in this optimized formulation, it effectively binds to the ergosterol in the fungal cell membrane. The use of nanocarriers enhances AmB’s stability by controlling drug molecule binding and slow release, indicating a potential alternative to LAmB. Further investigations and clinical validation are needed to confirm NLAmB’s role in treating CAM.
